# Facile synthesis of a novel Co–Ba–TPA MOF exhibiting nearly identical overpotential for electrochemical production of hydrogen and oxygen

**DOI:** 10.1039/d5ra06495c

**Published:** 2025-11-20

**Authors:** Promit Debnath, Ahmed Sharif, Shad Inquiad Mim, Md. Sahadat Hossain

**Affiliations:** a Department of Materials and Metallurgical Engineering, Bangladesh University of Engineering & Technology Dhaka-1000 Bangladesh asharif@mme.buet.ac.bd; b Pilot Plant & Process Development Centre, Bangladesh Council of Scientific and Industrial Research (BCSIR) Dhaka 1205 Bangladesh; c Institute of Glass & Ceramic Research and Testing, Bangladesh Council of Scientific and Industrial Research (BCSIR) Dhaka-1205 Bangladesh saz8455@gmail.com

## Abstract

In this study, the successful synthesis and comprehensive characterization of a novel cobalt–barium–terephthalic acid metal–organic framework (Co–Ba–TPA MOF) as a high-performance bifunctional electrocatalyst is reported. The prepared MOF exhibits synergistic interactions between Co and Ba metal centers within the terephthalic acid framework to exhibit an optimized electronic environment for enhanced electrocatalytic performance. Structural characterization through XRD, SEM and FTIR confirmed successful framework formation, while electrochemical evaluation revealed exceptional bifunctional activity with almost identical overpotential across both water splitting half-reactions. The MOF demonstrates an overpotential of 414.5 mV and 412.6 mV at 10 mA cm^−2^ for HER and OER maintaining a Tafel slope of 145.23 mV dec^−1^ and 106.18 mV dec^−1^, respectively. The OER performance is preserved up to higher current densities (*e.g.* 100 mA cm^−2^), which makes it possibly applicable for industrial applications. The almost identical overpotentials for both reactions represent a commendable achievement in bifunctional catalyst design by eliminating the typical trade-off between HER and OER activities shown by most single-metal catalysts and offering an alternative to expensive platinum-group metals while maintaining competitive electrocatalytic performance.

## Abstract 2

Co–Ba–TPA metal–organic framework demonstrates efficient bifunctional electrocatalytic performance for both hydrogen evolution reaction (HER, 414.5 mV) and oxygen evolution reaction (OER, 412.6 mV) in 1.0 M KOH alkaline electrolyte.

## Graphical abstract

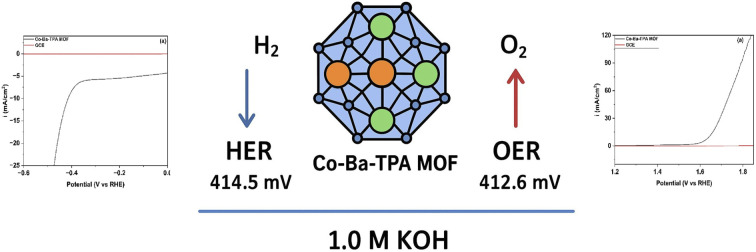

## 1. Introduction

The global search for sustainable and clean energy technologies has escalated the need for capable electrocatalysts fitted for water splitting to produce hydrogen. Among the myriad of electrocatalytic reactions, the hydrogen evolution reaction (HER) and oxygen evolution reaction (OER) represent the two fundamental half-reactions of water electrolysis, both requiring catalysts that can minimize energy barriers and enhance reaction kinetics.^1^ Presently, the advanced electrocatalysts for OER are ruthenium-based and platinum-based for HER.^2–4^ With a crustal abundance of <0.001 ppm and constrained reserves, Ru's scarcity poses critical supply challenges for water splitting technologies.^5,6^ While platinum-based materials have been considered as the benchmark for HER owing to their exceptional activity and low overpotentials, OER activity requires high overpotentials which limits the OER performance, making them unsuitable for bifunctional applications. Combined with their scarcity, high cost and restricted earth abundance have necessitated the exploration of alternative catalytic systems.^7,8^

Metal–organic frameworks (MOF) have brought about a revolution offering unprecedented opportunities for designing efficient electrocatalysts through their unique structural features and tunable properties. MOF catalysts with great porosity, atomically dispersed metal centers and higher specific surface area are considered as possible electro-catalysts for HER and OER.^6,9–11^ The crystalline character of MOFs, which exhibits well-defined metal nodes connected by organic linkers, provides exceptional control over active site distribution, pore architecture and electronic properties that are crucial for electrocatalytic applications.^12,13^ Recent investigations have demonstrated that MOF-based electrocatalysts can achieve remarkable performances in both HER and OER. For HER and OER of the CoNi-MOF-74/MXene/NF reach a current density of 100 mA cm^−2^ at an overpotential of 256 mV.^14^ The versatility of MOF synthesis allocates precise control of metal composition, oxidation states and coordination environments, enabling rational design strategies to optimize electrocatalytic performance.

The addition of multiple metal species within MOF structures has opened new route for achieving superior electrocatalytic performance through synergistic effects between different metal centers.^15,16^ Bimetallic MOFs represent a significant advancement over their monometallic counterparts, offering enhanced electronic properties, modified binding energies and optimized reaction pathways for both HER and OER processes.^17^ The synergistic interactions between two metal ions in bimetallic frameworks can lead to electronic redistribution, charge transfer phenomena, and modified d-band centers that collectively contribute to the adsorption and desorption of reaction intermediates.^18,19^ Recent developments in bimetallic MOF synthesis have demonstrated remarkable improvements in electrocatalytic performance, for OER at 10 mA cm^−2^ current density with Co–Mn-MOFs exerts an overpotential of 289 mV, while Ni–Cu bimetallic systems to achieve 30 mA cm^−2^ current density the HER and OER overpotentials were 78 and 220 mV, respectively.^20,21^ The strategic selection of metal combinations allows fine-tuning of electronic structures, where electron-donating metals can enhance the activity of electron-accepting partners, leading to optimal binding energies for multiple reaction intermediates simultaneously.^22,23^

This work focuses on the synthesis, comprehensive characterization and electrocatalytic evaluation of a novel Co–Ba–TPA bimetallic MOF system which represent an innovative approach to bifunctional water splitting catalysis. The strategic combination of cobalt and barium within a terephthalic acid framework offers unique opportunities to explore synergistic effects between transition metal and alkaline earth metal centers, potentially leading to exceptional HER and OER performance through optimized electronic structures and reaction mechanisms. The selection of cobalt as the primary catalytic metal comes from its well-established redox chemistry and favorable binding properties for both hydrogen and oxygen intermediates.^24,25^ Barium was selected as the secondary metal because firstly Ba^2+^ possesses a large ionic radius (1.35 Å) relative to most transition metals, which may increase the average metal–metal separation and enlarges pore apertures in the MOF, thereby improving mass transport and facilitating access to catalytically active sites. Second, Ba's high electro-positivity may promote partial electron donation into the carboxylate-metal network, shifting the Co d-band center toward intermediate binding energies that optimize adsorption/desorption of HER/OER intermediates.^26,27^ Third, extensive studies on A-site Ba-doped perovskite oxides (ABO_3_) have shown that Ba incorporation enhances structural robustness and resistance to redox-induced degradation through stronger ionic Madelung interactions and lattice cohesion, yielding superior electrocatalytic stability under harsh conditions.^28^ Density functional theory investigations of Ba-doped SrZrO_3_ also demonstrate that Ba substitution reduces lattice strain and narrows bandgaps by introducing new electronic states, highlighting its role in electronic modulation and phase stabilization.^27^ Terephthalic acid serves as an ideal organic linker due to its rigid aromatic structure, excellent stability, and ability to facilitate efficient electron transport between metal centers.^29^

The comprehensive characterization strategy employed in this work encompasses multiple analytical techniques to provide detailed insights into structural features, electronic properties, surface characteristics, and electrocatalytic performance. This research adds to the fast-expanding knowledge on bimetallic MOF electrocatalysts while advancing the fundamental understanding of structure–activity relationships crucial for the efficient design of next-generation water splitting catalysts.

The development of productive, cost-effective and stable electrocatalysts remains paramount for the successful implementation of hydrogen economy technologies. Through systematic investigation of the Co–Ba–TPA bimetallic MOF system, this work aims to demonstrate the potential of earth-abundant materials in achieving high-performance water electrolysis, contributing to the global transition toward sustainable energy technologies and carbon neutrality goals.

## 2. Materials and methodology

### 2.1 Materials

Cobalt nitrate hexahydrate [Co(NO_3_)_2_·6H_2_O], barium nitrate Ba(NO_3_)_2,_ terephthalic acid (C_8_H_6_O_4_) purchased from E-Merck, Germany were the main precursors. Ethanol and DMF have been purchased from E-Merck Germany, were used in all experiments.

### 2.2 Synthesis of Co–Ba–TPA MOF

**Fig. 1:**
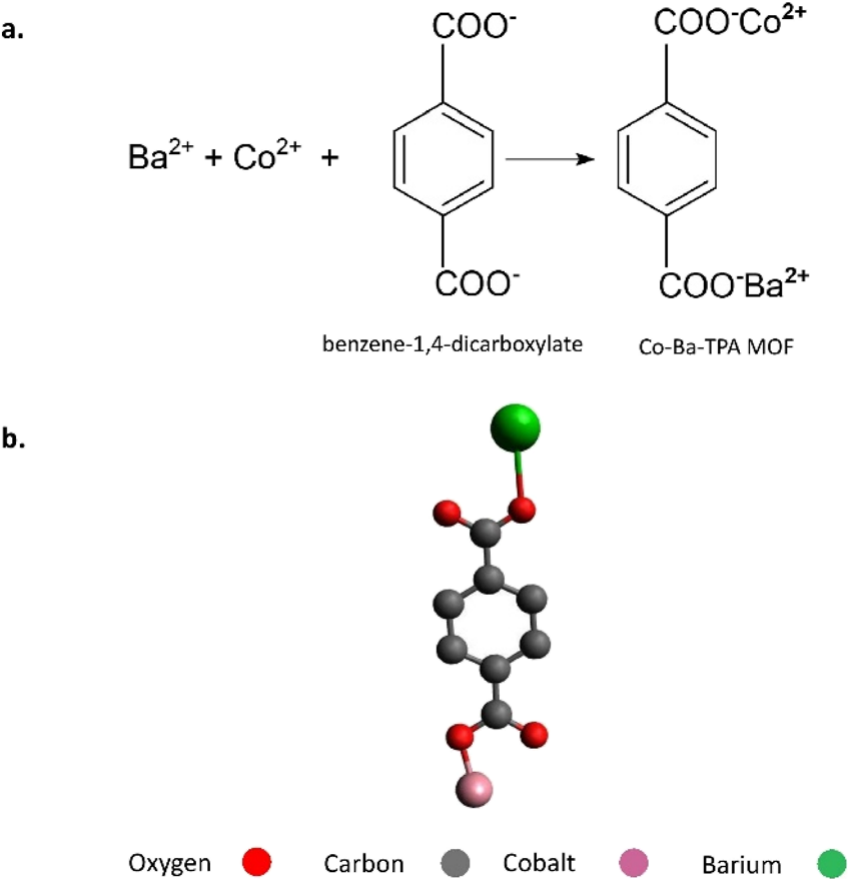
Schematic illustration of the conceptual reaction between Ba^2+^, Co^2+^ and terephthalic acid (a), highlighting the formation of Co–Ba–TPA coordination motifs (b). This simplified depiction is not meant to represent the actual extended MOF crystal structure or precise coordination geometries. For charge neutrality and spatial arrangement, actual MOF structures feature extended networks with carboxylate linkages between metal nodes.

1.5 mmol Co(NO_3_)_3_·6H_2_O, 1.5 mmol Ba(NO_3_)_2_ and 4.5 mmol terephthalic acid were mixed in 60 mL DMF and stirred until completely dissolved. This was taken to a 100 mL Teflon lined Stainless Steel (SS) autoclave and treated hydrothermally at 100 °C for 24 hours. After hydrothermal synthesis, the mixture was centrifuged at 6000 rpm for 10 min to collect the solid, which was then redispersed in 10 mL of ethanol and agitated for 15 min. The suspension was centrifuged again (6000 rpm, 10 min) to recover the solid, which was subsequently washed with 10 mL of DMF under identical conditions. This ethanol/DMF sequence was repeated twice more (totalling three ethanol washes and three DMF washes). After the final DMF wash and centrifugation, the recovered solid was transferred to a regular drying oven and dried at 40 °C overnight (Fig. 1).

### 2.3 Characterization

#### 2.3.1 XRD

Powder XRD patterns were recorded on a Rikagu SE diffractometer using Cu Kα radiation (*λ* = 1.5406 Å) at 40 kV and 50 mA. Over a 2*θ* range of 10–70°, data were acquired at 0.01° step size. Suppression in β-radiation was achieved by a nickel filter, and peak calibration system relied on silicon standard. The detector had been blocked with a nickel shield to prevent beta radiation from the X-ray source, and silicon reference was calibrated against before analysis.

#### 2.3.2 SEM

A Tescan Vega Compact Scanning Electron Microscope was used for the morphological and structural analysis of synthesized MOF.

#### 2.3.3 FTIR

A Shimadzu IR-Prestige 21 FTIR spectrometer was used to record infrared spectra, equipped with an ATR. FTIR spectrum was recorded with 400 and 4000 cm^−1^ resolution using 30 scans per sample at 25 °C, 60% relative humidity under laboratory conditions. A spectral resolution of 4 cm^−1^ was used for the characterization.

#### 2.3.4 Electrochemical measurement

Electrochemical experiments were carried out with a CS300 model instrument of the Corrtest manufacturer (China) electrochemical analyzer. A standard three-electrode system (Ag/AgCl electrode as the reference electrode, Pt as the counter electrode and prepared glassy carbon (GC) as the working electrode) was used. GC electrode was modified using the prepared MOF as an active material. To prepare the working electrode, 10 mg of catalyst was transferred into 1.5 mL acetone and sonicated for 30 min to ensure uniform dispersion. 10 µL of catalyst ink was drop-casted on 0.07 cm^2^ of glassy carbon electrode, which was slowly dried at room temperature to form the working electrode. The final mass loading on the working electrode was 0.96 mg cm^−2^. The OER and HER performances were characterized in 1.0 M KOH using the linear sweep voltammetry (LSV) with a scan rate of 25 mV s^−1^ at 25 °C. 30 cycles of CV had been conducted before measurement; this was essential for the activation of the electrode materials. The concentration of KCl used in Ag/AgCl reference electrode was 3.5 M. The potentials were converted to the reversible hydrogen electrode (RHE) according to the following eqn (1):

1 *E*_RHE_ = *E*_Ag/AgCl_ + 0.197 + 0.0591 × pH

And the overpotentials (*η*) for HER and OER were calculated according to the formula (2) and (3):

2 *η*_HER_ = *E*_RHE_

3 *η*_OER_ = *E*_RHE_ −1.23 V

## 3. Results and discussion

### 3.1 Structural analysis

**Fig. 2:**
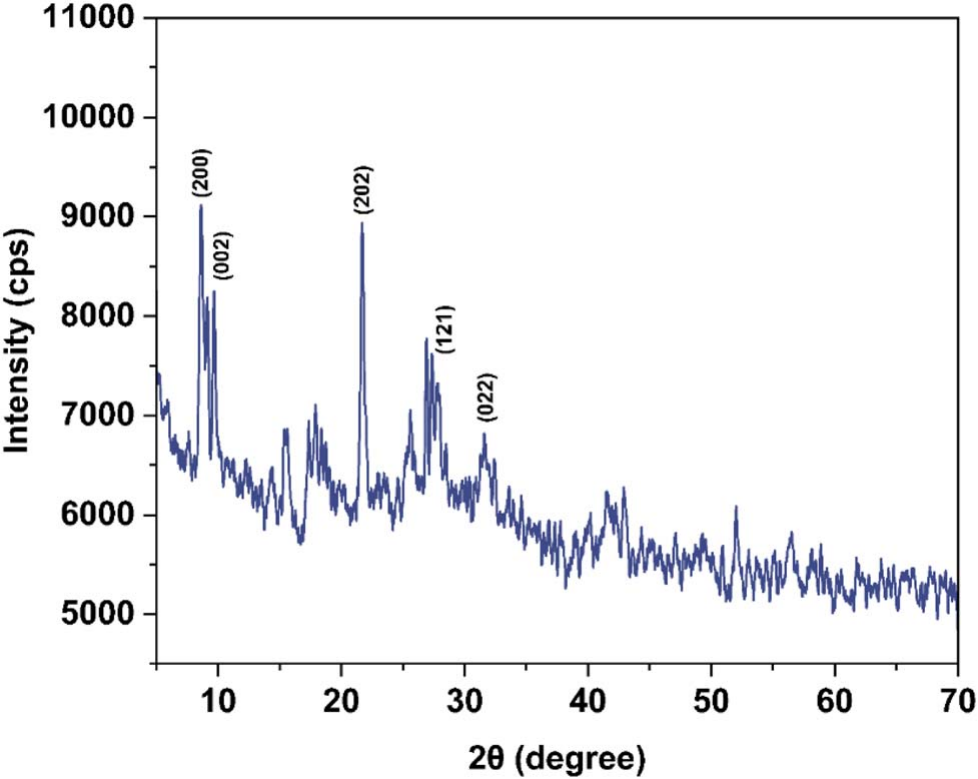
XRD Pattern of Co–Ba–TPA MOF.

The X-ray diffraction pattern of synthesized Co–Ba–TPA bimetallic metal organic framework is illustrated in Fig. 2. Seven major diffraction peaks were observed at 2*θ* values of 8.614°, 9.700°, 21.703°, 27.39°, 31.56°, 52.022° and 56.38° with corresponding *d*-spacing values of 10.257, 9.111, 4.092, 3.253, 2.832, 1.7565 and 1.631 Å, respectively. The most intense peak appeared at 2*θ* = 27.39°, followed by significant peaks at 8.614° and 21.703°. The presence of low-angle reflections at 8.614° and 9.700° with large *d*-spacing values (>9 Å) is characteristic of MOF materials, indicating the formation of an ordered porous framework structure with extended crystalline periodicity.^30^ The observed *d*-spacing values confirmed the formation of ordered arrangement of metal nodes connected by terephthalic acid linkers, forming a three-dimensional porous network.^31,32^ The sharp, well-defined peaks with relatively narrow full-width-at-half-maximum (FWHM) values ranging from 0.10° to 1.69° demonstrated good crystallinity of the synthesized material. The calculated crystallite sizes range from approximately 4.8 to 88.4 nm for different reflections, suggested nanoscale crystalline domains typical of MOF materials.^33^ Crystallite size, microstrain and dislocation density were calculated using eqn (4)–(6).4
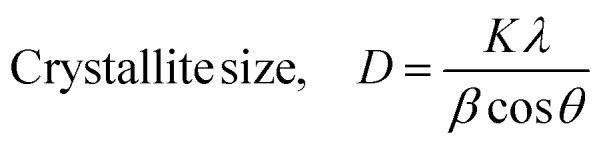

5 Microstrain, *ε* = *β*/4 tan *θ*

6 Dislocation density, *δ* = 1/*D*^2^

where, *K* = 0.9 is the shape factor, *λ* = 1.5406 Å (Cu Kα radiation), *β* in radian is the peak width at half maximum (FWHM) and *θ* is the Bragg angle. The crystallographic parameters are listed in Table 1.

**Table 1:** Crystallographic parameters of Co–Ba–TPA MOF using Debye–Scherrer equation

2*θ* (°)	*d* (Å)	Plane	FWHM (°)	Crystallite size, *D*_c_ (nm)	Dislocation density, *δ*	Microstrain, *ε*
8.614	10.257	200	0.32	24.90	1.61	0.018
9.7	9.111	002	0.121	65.89	0.23	0.006
21.703	4.092	202	0.21	38.52	0.67	0.005
27.39	3.253	121	1.69	4.84	42.72	0.030
31.56	2.832	022	0.5	16.51	3.67	0.008
52.022	1.7565	229	0.1	88.40	0.13	0.001
56.38	1.631	351	0.46	19.59	2.60	0.004
Average				36.95 ± 27.73	7.38	0.010

The comparative XRD analysis of Co–Ba–TPA bimetallic MOF against both experimental and computational monometallic Co–TPA and computational Ba–TPA revealed distinctive crystallographic features confirming successful bimetallic framework formation. The low-angle peak at 8.614°, which exhibited the second highest intensity, showed satisfactory agreement with existing experimental literature on Co–TPA MOF. The peak at 21.703° and 31.56° also matched approximately well with the CCDC card no: 1430631, which is for (µ-terephthalato)-(µ-*N*,*N*-dimethylformamide)-cobalt.^34,35^ The CCDC: 130804 is for (µ_7_-terephthalato)-barium *i.e.* Ba–TPA with orthorhombic structure.^36^ The low-angle MOF-characteristic peak at 9.70° (*d* = 9.111 Å) correspond closely to it at 9.35° (*d* = 9.447 Å), indicating retention of both metal coordination environments within the bimetallic framework. The peak at around 52° and 56° closely matched with this computational result. The most intense peak at 27.39° (*d* = 3.253 Å) appears intermediate between Co–TPA (26.98°, *d* = 3.302 Å) and Ba–TPA (28.57°, *d* = 3.121 Å) computational predictions, demonstrating the synergistic structural modification arising from Co–Ba heterogeneous metal nodes rather than simple phase segregation. The diffraction pattern is consistent with bimetallic MOF formation, where the two metal ions are coordinated within the same crystalline framework rather than existing as separate phases.^37,38^ The systematic peak shifts of 0.3–1.0° relative to monometallic controls validate the electronic and structural perturbations expected from heterogeneous Co^2+^–Ba^2+^ coordination environments, supporting the proposed bimetallic MOF architecture essential for enhanced electrocatalytic performance. The crystallographic planes of observed diffraction peaks have been incorporated in Table 1 using CCDC: 130804 and 1430631 for Ba–TPA and Co–TPA, respectively.

### 3.2 Morphological analysis

**Fig. 3:**
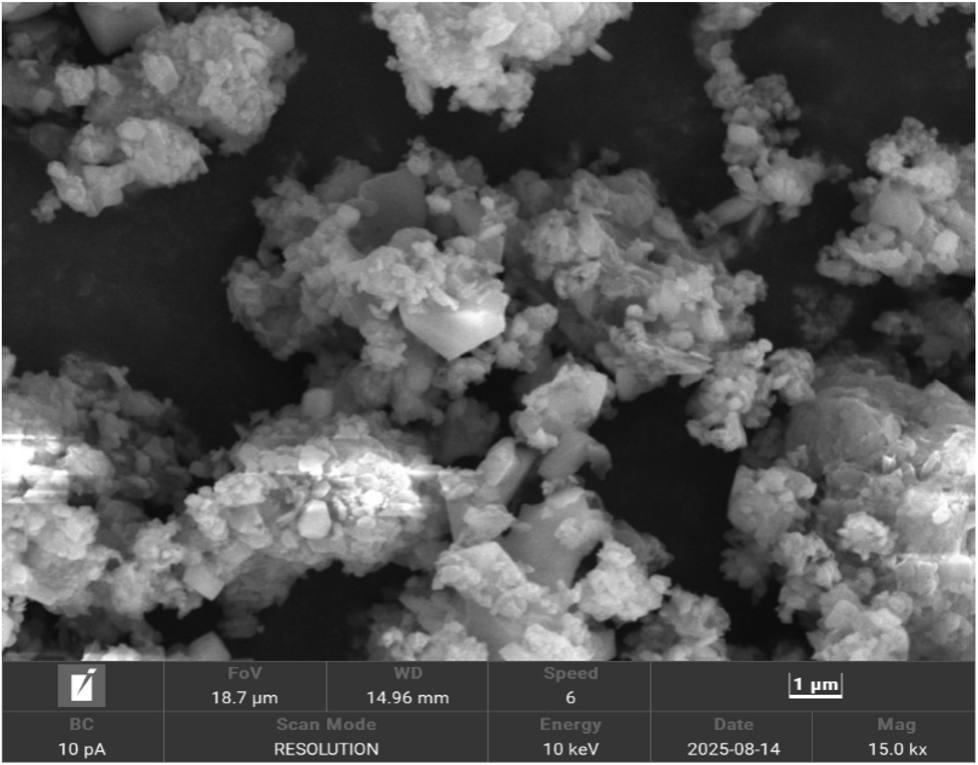
SEM image of Co–Ba–TPA MOF.

The SEM image of synthesized Co–Ba–TPA MOF is shown in Fig. 3. The material is composed of dense, chain-like agglomerated crystallites where distinct plate-like and blocky structure can be observed. The rough and porous surface texture of these agglomerates is consistent with the presence of interparticle void, which is a key characteristic of MOF. The co-existence of crystallite domains and hierarchical aggregation provides high density of accessible active sites, which are favourable for mass transport and charge transfer. The observed morphology is anticipated to enhance the catalytic application of the Co–Ba–TPA MOF.

### 3.3 FTIR analysis

**Fig. 4:**
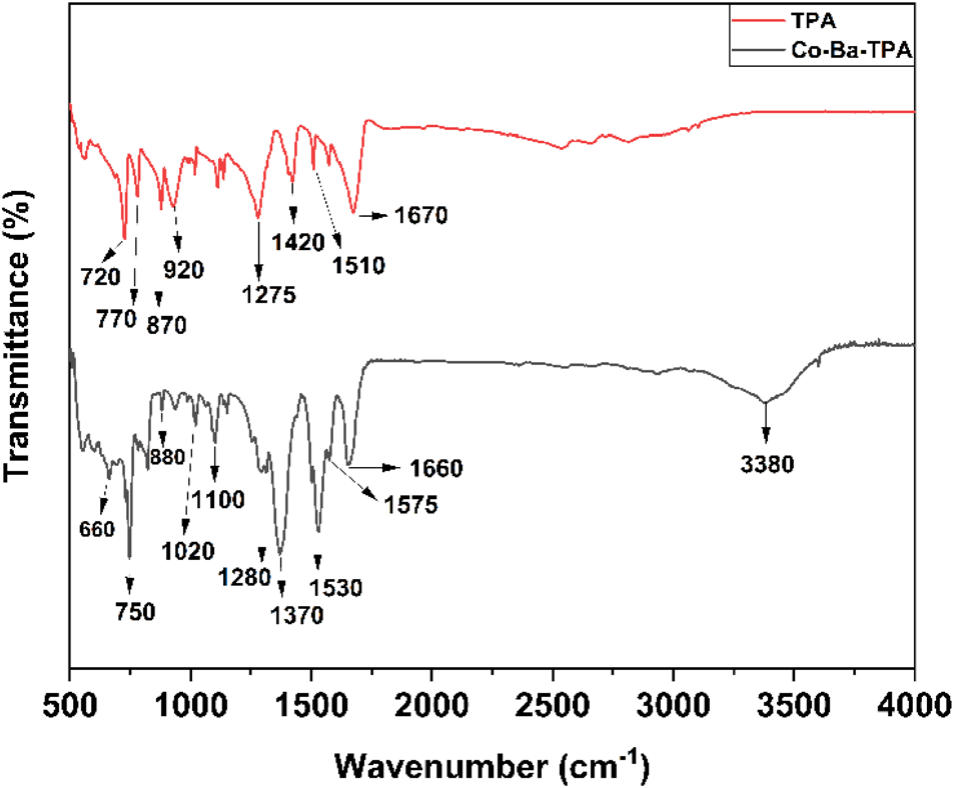
FTIR Spectrum of Co–Ba–TPA MOF.

The FTIR spectrum of both TPA and Co–Ba–TPA MOF is illustrated in Fig. 4. For Co–Ba–TPA, the broad band at 3380 cm^−1^ corresponds to O–H stretching, indicating adsorbed water. Key evidence for metal–ligand coordination is seen in the asymmetric COO^−^ stretch at 1660 cm^−1^ and symmetric stretch at 1530 cm^−1^, both notably shifted from the TPA (1670 cm^−1^ and 1510 cm^−1^, respectively), confirming effective coordination to Co and Ba centers. This peak shift is consistent with coordinated carboxylate groups in MOF structures, where the electron density redistribution upon metal coordination affects the vibrational frequency.^32,39^ Notably, the distinct band at 1370 cm^−1^ is attributed to secondary carboxylate vibrations, possibly indicating the coexistence of different coordination modes – bridging, chelating or a mixture thereof. This is plausible given the combination of Co^2+^ and Ba^2+^ ions in the MOF, whose differing preferences can generate both bridging and chelating ligand environments. The observed Δ*ν* = 130 cm^−1^ supports a substantial bridging component, but the overall spectral profile suggests a mixed or intermediate coordination environment.^40,41^ Throughout the aromatic region, in-plane C=C stretching vibrations manifest at 1575 cm^−1^ for MOF, slightly shifted from their free-linker positions (1510–1420 cm^−1^), revealing π-system perturbation by metal coordination. The sharp peak at 1370 cm^−1^ at MOF, is due to secondary carboxylate vibration. A combined aromatic ring breathing and symmetric carboxylate deformation band at 1425 cm^−1^ further corroborates the bridging coordination environment in the MOF, while the C–O stretch of the coordinated carboxylate at 1280 cm^−1^ – shifted upward from ∼1275 cm^−1^ in the free acid – attests to the metal–oxygen bond character.^42^ In the fingerprint region (1160–650 cm^−1^), multiple in-plane aromatic C–H bending and skeletal C–C vibrations (notably near 1100 and 1020 cm^−1^) confirm the *para*-disubstituted benzene framework and sharp out-of-plane C–H bending modes at 940 cm^−1^, 880 cm^−1^ and 750 cm^−1^ validate the terephthalate substitution pattern. Finally, broad but identifiable lattice vibrations in the 700–650 cm^−1^ range provide direct evidence of Co–O and Ba–O bonds within the heterometallic nodes.^43,44^

### 3.4 HER performance and mechanism

**Fig. 5:**
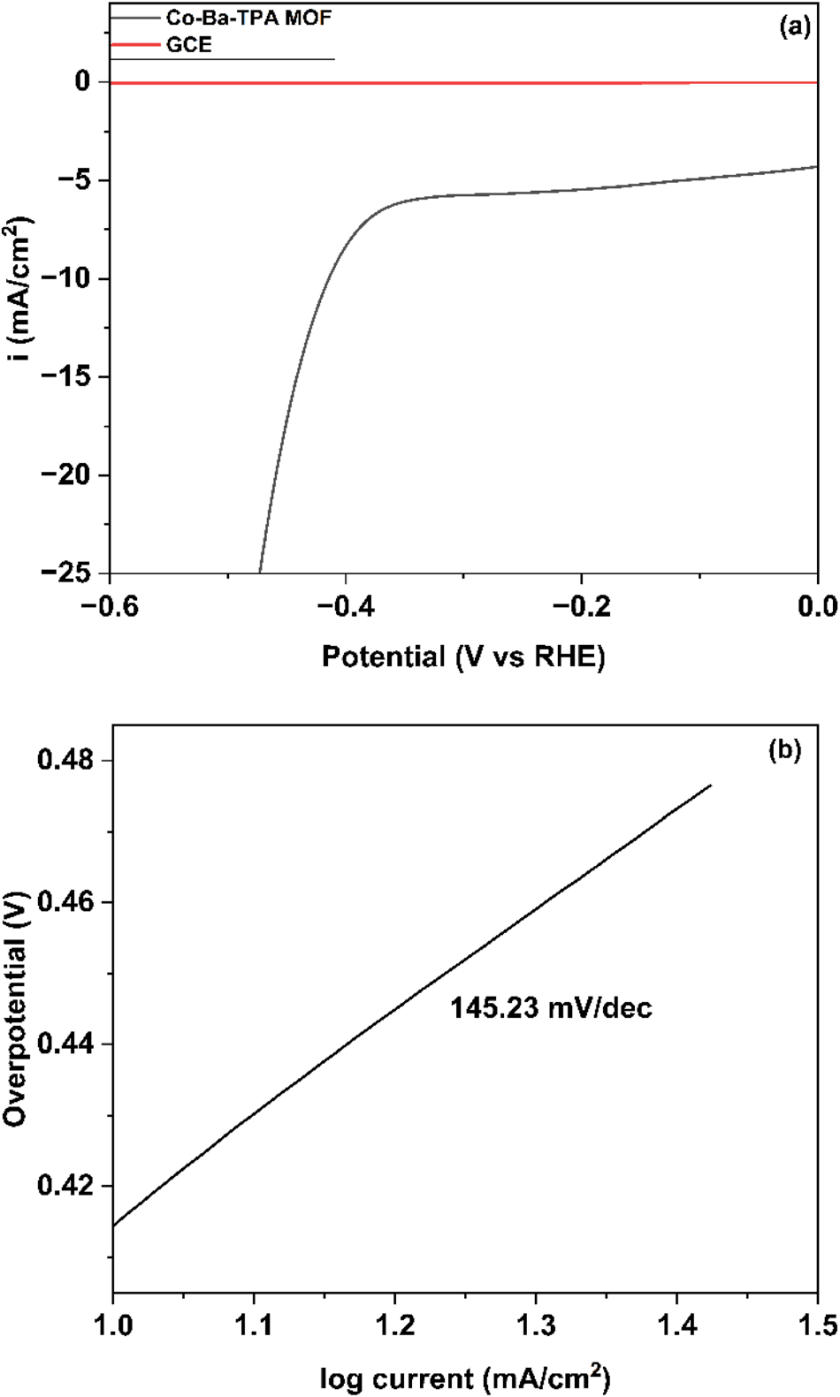
(a) Polarization curve (b) Tafel plot of Co–Ba–TPA MOF for HER.

The polarization curve (Fig. 5a) reveals that the Co–Ba–TPA MOF exhibits an overpotential of 414.5 mV to accomplish a current density of 10 mA cm^−2^, which represents a benchmark current density for practical electrocatalytic applications. The MOF exhibits a current density of 5.45 mA cm^−2^ at 0.2 V potential. The calculated onset potential for HER is 0.68 V *vs.* RHE (at 1 mA cm^−2^), indicating the potential at which hydrogen evolution becomes kinetically favorable.

The HER reactions that occur in alkaline medium are presented in eqn (7)–(9):^45^

7 Volmer step: H_2_O + e^−^ → H* + OH^−^

8 Heyrovsky step: H_2_O + e^−^ → H_2_ + OH^−^ (or)

9 Tafel step: 2H* → H_2_

* means an active center on the electrocatalyst.

Based on the coverage of H_ads_, the mechanism by which HER proceeds are studied. If adsorbate site coverage of H_ads_ is low, then H_ads_ reacts with a proton and electron at the same time because neighbouring sites are available near the adsorbed hydrogen that lead to Heyrovsky reaction. When the local surface coverage is very large, H_2_ is formed if two adjacent H_ads_ reunite. If Volmer step is the rate-limiting step in HER, the expected Tafel slope is approximately 120 mV dec^−1^ (sometimes reported as 118–120 mV dec^−1^). For Heyrovsky and Tafel step the slopes are approximately 40 mV dec^−1^ and 30 mV dec^−1^, respectively.^46–48^

Based on Fig. 5a, the corresponding Tafel plot is illustrated in Fig. 5b. Tafel slope (*b*) is an essential parameter in HER, which is determined by fitting the experimental polarization data to the Tafel equation. The measured Tafel slope of 145.23 mV dec^−1^, which is very close to the theoretically expected Tafel slope, suggests that the Volmer step is the rate-determining step in the alkaline HER process. This relatively high Tafel slope indicates that the initial adsorption of hydrogen intermediates onto the active sites is kinetically limiting. The exchange current density (*j*_0_) was calculated to be 1.40 × 10^−2^ mA cm^−2^ using the Tafel equation,^49^*η* = *b* log(*j*/*j*_0_), where *j*_0_ is the exchange current density, reflecting the intrinsic catalytic activity of the MOF material for hydrogen evolution.^45,46^ Barium's high electro-positivity may induce electron density redistribution toward cobalt centres, which could optimize cobalt's d-band centre and favor hydrogen adsorption thermodynamics. The Co–Ba pair may form bifunctional sites where barium potentially promotes water dissociation (Volmer step) while cobalt facilitates hydrogen adsorption/desorption (Heyrovsky/Tafel steps). Coordination with TPA ligands could lead to Co–O, Ba–O, and Co–Ba–O clusters, possibly fine-tuning electronic properties and enhancing catalytic water activation.

### 3.5 OER performance and mechanism

**Fig. 6:**
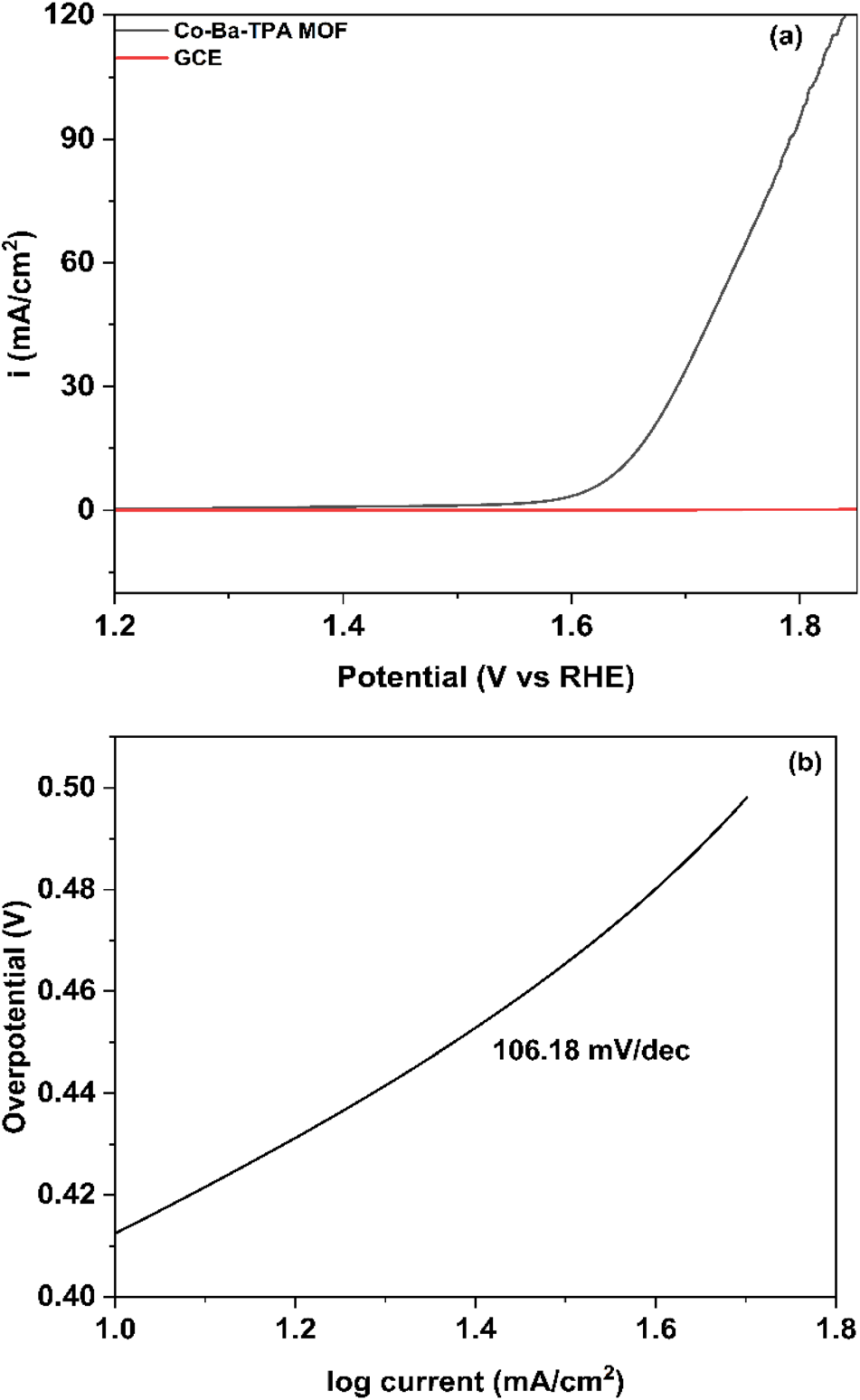
(a) Polarization curve (b) Tafel plot of Co–Ba–TPA MOF for OER.

The potential of our synthesized MOF for OER is scrutinized next. Fig. 6a illustrates the polarization curve and Fig. 6(b) the corresponding Tafel plot. For the OER, Co–Ba–TPA MOF demonstrates superior performance compared to its HER activity. The OER polarization curve shows an overpotential of 412.6 mV at 10 mA cm^−2^, which is remarkably close to the HER overpotential, indicating balanced bifunctional electrocatalytic properties. The bare GCE showed negligible OER activity in the same condition, which made sure the OER activity is indeed from the synthesized MOF. The calculated onset potential for OER is 1.45 V *vs.* RHE (at 1 mA cm^−2^), representing the thermodynamic threshold for oxygen evolution to become kinetically accessible. At 100 mA cm^−2^, the potential is 1.8 V *vs.* RHE, which makes it potentially accessible for industrial application.

The four-electron pathway for OER under alkaline condition are reported to undergo according to the following eqn (10)–(13):^45^

10 * + OH^−^ → OH* + e^−^

11 OH* + OH^−^ → O* + H_2_O + e^−^

12 O* + OH^−^ → OOH* + e^−^

13 OOH* + OH^−^ → O_2_ + H_2_O + e^−^

The * represents the active site when OER occurs. The OH*, O*, OOH* represents the intermediate species that are adsorbed on the active sites. Tafel plot was used to evaluate the kinetics of OER. The Tafel slope of 106.18 mV dec^−1^ provides mechanistic insights into the four-electron oxygen evolution process. This value suggests that a chemical step following the first electron transfer is rate-determining, consistent with the formation of metal–oxo intermediates or the coupling of oxygen-containing adsorbates.^50^ The lower Tafel slope compared to HER indicates more favourable electron transfer kinetics for the OER process on this MOF catalyst. The calculated exchange current density for OER is 1.30 × 10^−3^ mA cm^−2^, which is lower than that of HER, reflecting the inherently more complex four-electron nature of the oxygen evolution process.^45,51^ The OER performance of Co–Ba–TPA MOF can be attributed to the unique electronic structure created by the bimetallic Co–Ba nodes within the terephthalic acid framework. The cobalt centers likely serve as the primary active sites for oxygen evolution, while the barium incorporation may modulate the electronic environment to optimize the binding energies of oxygen-containing intermediates (O*, OH*, OOH*). Important parameters for HER and OER are tabulated in Table 2.

**Table 2:** Electrochemical parameters of HER and OER

Parameters	HER	OER
Onset potential at 1 mA cm^−2^	0.68 V *vs.* RHE	1.45 V *vs.* RHE
Overpotential at 10 mA cm^−2^ (*η*_10_)	414.5 mV	412.6 mV
Tafel slope (*b*)	145.23 mV dec^−1^	106.18 mV dec^−1^
Exchange current density (*j*_0_), mA cm^−2^	1.40 × 10^−2^	1.30 × 10^−3^

**Fig. 7:**
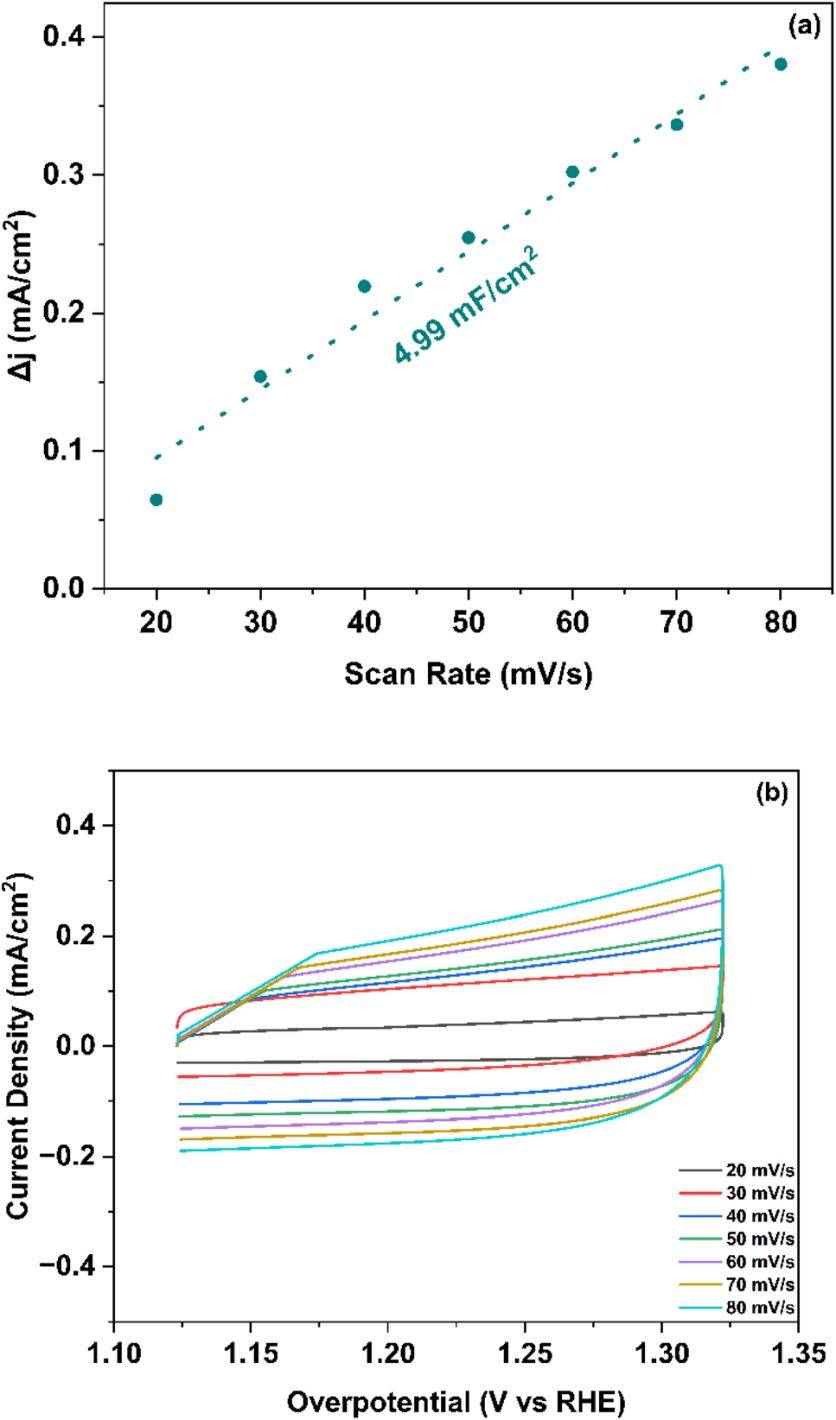
(a) Δ*j* plot as a function of scan rate and *C*_dl_ calculation (b) CV curves at various scan rates.

The electrochemical double-layer capacitance (*C*_dl_) measurement revealed a substantial value of 4.99 mF cm^−2^, as depicted and calculated from Fig. 7a and b. This high *C*_dl_ value corresponds to an electrochemically active surface area (ECSA) of 124.8 cm^2^, calculated using equation, ECSA = *C*_dl_/*C*_s_. The specific capacitance (*C*_s_) was taken as 0.040 mF cm^−2^ in 1.0 M KOH.^52^ This value indicates extensive exposure of active sites within the Co–Ba–TPA framework structure. The large ECSA directly correlates with the enhanced bifunctional electrocatalytic performance, as shown by the synthesized MOF. The high double layer capacitance suggests effective ion accessibility and charge transfer at the electrode–electrolyte interface, while the substantial ECSA provides numerous active sites for both hydrogen and oxygen evolution reactions. The correlation between high ECSA (124.8 cm^2^) and moderate overpotentials validates the structure–activity relationship in this bimetallic MOF system, where increased surface area directly translates to improved catalytic performance for water splitting applications.

**Fig. 8:**
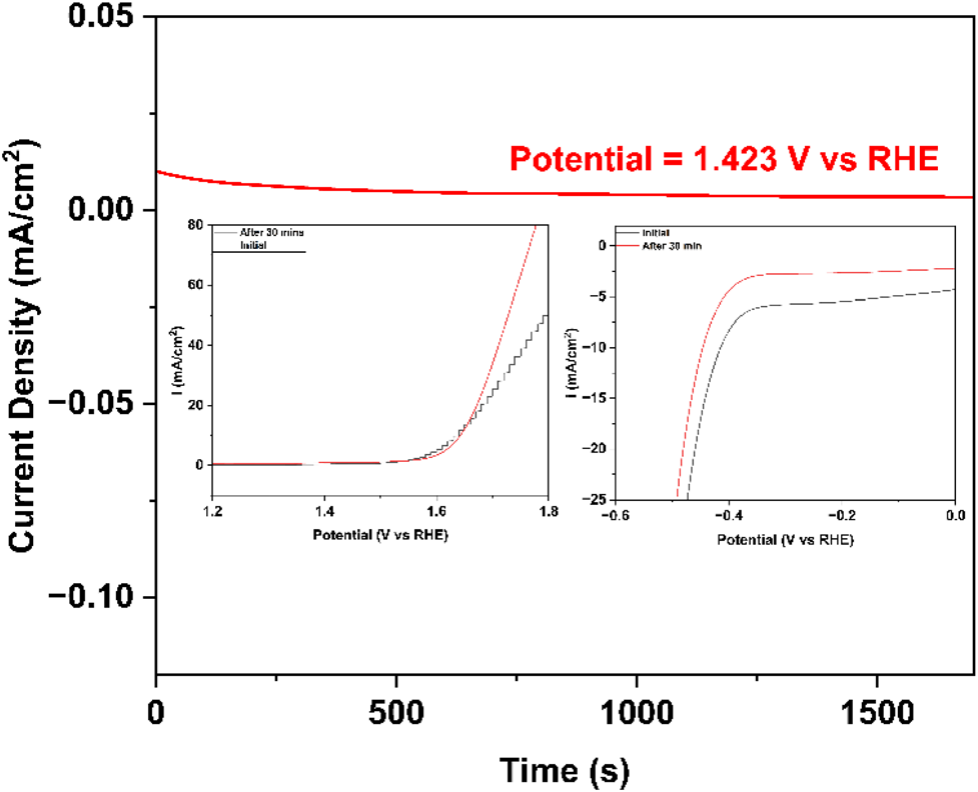
Stability for Co–Ba–TPA MOF using chronoamperometry at 1.423 V *vs.* RHE and the inset is the LSV curves before and after 30 min test.

To measure the stability, chronoamperometry was executed at 1.423 V *vs.* RHE for 30 minutes, illustrated in Fig. 8. The LSV curves before and after 30 min evaluation were also added in the figure. After 30 minutes evaluation, the current density shows 67% retention.

## 4. Discussion

The one-step solvothermal synthesized novel Co–Ba–TPA MOF was characterized using XRD, SEM and FTIR. The electrochemical experiment was conducted in 1.0 M KOH. The MOF exhibited a well-defined bimetallic crystalline framework (4.8–88.4 nm crystallite size) with low-angle XRD peaks at 8.614° and 9.700°, confirming an ordered porous architecture and slight peak shifts (0.3–1.0°). SEM revealed chain-like, porous agglomerates, while FTIR bands verified mixed mode of coordination within the framework. The high ECSA (124.8 cm^2^; *C*_dl_ = 4.99 mF cm^−2^) correlated directly with electrocatalytic performance. The synthesized MOF exhibits overpotentials (414.5, 412.6 mV for HER and OER, respectively) which are higher than the comparative systems listed in Table 3. However, a critical distinction exists between the systems: the reported high-performance MOFs are composite materials or modified frameworks that have undergone extensive post-synthetic modifications, which fundamentally alter their properties compared to pristine MOFs. For instance, nanoflakes of nitrogen doped C@NiFeP was derived from MOF (162 mV HER, 270 mV OER) involve multi-step pyrolysis at high temperatures (typically 350–500 °C), phosphidation processes using NaH_2_PO_2_ and heteroatom doping strategies. Similarly, MXene-containing MOF-derived CoS_2_/C nanocomposites (270 mV HER, 257 mV OER) require incorporation of two-dimensional MXene nanosheets through multi-step synthesis followed by sulfidation and carbonization. The significantly lower overpotentials shown by these systems can be due to three key factors absent in pristine MOF structures. Firstly, enhanced electrical conductivity – pyrolysis changes MOFs into highly conductive carbon matrices embedded with metal phosphide/sulfide nanoparticles that helps rapid electron transfer and reduces charge transfer resistance.^53^ Secondly, chemical transformation processes (phosphidation, sulfidation, high-temperature carbonization) alter coordination environments and create active sites with optimal binding energies for H* (HER) and *OH/*O/*OOH (OER) intermediates, approaching volcano plot apex activities.^54^ Finally, MOF-derived porous carbon structures with hierarchical porosity facilitate superior electrolyte penetration, gas bubble release and ion diffusion which reduces mass transport limitations particularly at higher current densities.^55^ Nevertheless, this Co–Ba–TPA MOF represents the first reported cobalt–barium terephthalic acid framework synthesized using a simple one-step solvothermal process (100 °C, 24 h) without any post-synthetic modifications, pyrolysis or composite formation. Importantly, while the absolute performance is modest compared to extensively engineered composites, the synthesized framework exhibits nearly identical overpotentials for HER (414.5 mV) and OER (412.6 mV) – a difference of only 1.9 mV at 10 mA cm^−2^. This balanced bifunctionality is highly desirable for practical water electrolysis systems, as most bifunctional catalysts show significant trade-offs between HER and OER activities, like the catalysts reported in Table 3. This finding makes this MOF a foundation for future modifications and demonstrates the potential of mixed Co–Ba systems for electrocatalytic applications.

**Table 3:** Performance comparison of the synthesized MOF with recently reported MOF systems

MOF system	*η*_10_ for HER (mV)	*η*_10_ for OER (mV)	Electrolyte	Reference
Nanoflakes of nitrogen doped C@NiFeP was derived from MOF	162	270	—	56
2D NiFe-MOF-74 nanosheets	195	208	—	57
Fe-doped Co-based MOF	150	—	1.0 M KOH	58
M-NC-CoCu	240	310	1.0 M KOH	59
MOF-based FC-NC/NC	193	295	1.0 M KOH	60
CoS@C/MXene	270	257	1.0 M KOH for OER and 0.5 M H_2_SO_4_ for HER	61
Co–Ba-TPA MOF	414.5	412.6	1.0 M KOH	This work

## 5. Conclusion

In summary, a novel cobalt–barium terephthalic acid MOF (Co–Ba–TPA) was synthesized in a single, facile step and shown to deliver balanced bifunctional water-splitting activity, with nearly identical HER and OER overpotentials of 414.5 and 412.6 mV at 10 mA cm^−2^. Its Tafel slopes of 145.2 mV dec^−1^ (HER) and 106.2 mV dec^−1^ (OER) underscore favorable kinetics. The synergistic electronic interactions between cobalt and barium metal centers, combined with the inherent advantages of the MOF structure contribute to the superior electrocatalytic performance observed in this system. Importantly, the utilization of earth-abundant cobalt and barium elements presents a potential cost-effective and industrially scalable alternative to precious metal catalysts, offering significantly reduced environmental impact compared to noble metal extraction and processing, while achieving performance metrics that are moderate compared to many reported precious metal-based systems. One drawback of this reported system is the lack of the comparison figure containing simulated and experimental patterns of Co–Ba–TPA MOF, adding that would've added more depth to the reported structure. The Co–Ba–TPA MOF establishes a new paradigm for designing bimetallic frameworks that achieve balanced HER/OER performance and paves the way for further optimization-including the exploration of other earth-abundant bimetallic combinations within various MOF frameworks, optimization of synthesis parameters to enhance crystallinity and porosity, development of supported catalyst architectures for improved electrical conductivity, investigation of reaction mechanisms through advanced *in situ* characterization techniques and comprehensive techno-economic analysis of scaled manufacturing processes. The findings contribute meaningfully to the growing body of knowledge in sustainable energy conversion, electrocatalysis and materials chemistry, ultimately representing a promising and significant step toward achieving large-scale, economically viable hydrogen production infrastructure that will be essential for the global transition to a carbon-neutral energy economy and the mitigation of climate change impacts.

## Conflicts of interest

There are no conflicts to declare.

## Data Availability

Data will be made available on request.
